# The general public new views on deceased organ donation in China

**DOI:** 10.1097/MD.0000000000023438

**Published:** 2020-12-11

**Authors:** Xiaoshan Li, Junyan Miao, Rong Gao, Di Hu, Gongtao Qian, Dong Wei, Jianmei Zhou, Lihua Zhang, Weiwei Xu, Jingyu Chen, Chunxiao Hu

**Affiliations:** a Department of Lung Transplant Center, the Affiliated Wuxi People's Hospital of Nanjing Medical University, Wuxi; b School of Public Health, Nantong University, Nantong; c Department of Clinical Laboratory, Wuxi People's Hospital Affiliated to Nanjing Medical University, Wuxi, Jiangsu, China.

**Keywords:** deceased organ donation, impact factor, systematic review, transplantation, view

## Abstract

**Background:**

The organ donation in China has developed rapidly since fully launched donations after citizens death in 2015. This study was conducted to evaluate how the Chinese general public views changed on deceased organ donation, and to improve the donation process.

**Methods:**

A total of 110 eligible studies, including 103, 410 individuals, were selected to analyze through searching PubMed, CBMdisc, CQVIP, CNKI, and Wanfang Data from Jan 1, 1990 to May 31, 2019. The pooled proportions (and 95% CIs) of cognition, attitudes and willingness related to organ donation were calculated using the Freeman–Tukey double arcsine transformation.

**Results:**

The pooled proportions of knowing about organ donation and willing to donate increased from 84.6% (73.0–93.4) and 32.4% (23.9–41.6) before 2015, to 86.4% (74.5–95.1) and 39.9% (32.8–47.2) after 2015, respectively. The willingness to posthumous organ donation for cornea, heart, kidney, and liver had a significant improvement. Especially, the proportion of willingness to donate cornea increased to 56.0% (43.3–68.3) after 2015, from 39.2% (31.2–47.4) before 2015. However, although 69.7% (62.7–76.4) of participants approved the deceased organ donation, only 35.6% (29.7–41.8) and 43.9% (37.2–50.8) were willing to donate their own and relatives organs postmortem, respectively. The leading reasons for refraining from donating organs postmortem were distrusting the medical professionals (49.8%, 35.2–64.4) and traditional Chinese values (40.6%, 32.4–49.0). Popularizing knowledge about organ donation (61.5%, 45.7–76.1), humanitarian aid (57.1%, 48.8–65.3), and priority of using donated organs for relatives (53.1%, 30.8–74.7) were the applauded strategies to improve the willingness to posthumous organ donation.

**Conclusions:**

The willingness toward posthumous organ donation has a significant improvement among Chinese general public since 2015, however, several important measures still need to be taken to promote the favorable attitudes and willingness toward organ donation.

## 1 Introduction

Organ transplantation is an effective treatment for end-stage organ dysfunction, while organ donation is the premise and foundation of transplantation. Deceased organ donation widely advocated in the transplanting field is consistent with internationally recognized standards of “dead, voluntary and gratuitous”.^[1]^ Critical shortage of donor organs and disparity between the supply and demand for organ transplantation are common issues all over the world. Deceased organ donation is developing rapidly worldwide, especially in European and American countries. Spain and Croatia have the highest posthumous organ donation rates in the world, and the donation rate of per million population (PMP) was above 39.7 in 2015.^[2,3]^ The United States is the first country in the world to carry out organ transplantation, and with the largest number of donations. Its PMP has remained stable at over 25.0 in the past decade and the donation number nearly increases to 16, 000 in 2016.^[4]^ In addition, British and Italian's PMP are also over 20.0 currently.^[2]^

After more than 10 years of exploration and reform, the posthumous organ donation in China has made a great progress. In 2007, the State Council promulgated the Regulations on Human Organ Transplantation, which has led to the legalization of organ donation and transplantation in China. Since China fully launched donations after citizens death (DCD) in 2015, posthumous organ donation has become the only source of organ transplantation in China. The number of organ donations and PMP reached 2766 and 2.01 in 2015, respectively, which was 67 times higher than that in 2010, the time of launched the pilot project of organ donation in China.^[5]^ Until the end of May, 2019, the number of donated individuals and organs have increased to 23, 574 and 67, 277, respectively, in China, thus China has become the second-largest donor country in the world.^[6]^ In addition, more than 1, 257, 049 Chinese citizens have registered as organ donation volunteers, by far.^[6]^ Nevertheless, the China's PMP is only approximately 4.5 in 2018, which is substantially below the levels of European and America.^[7]^ It is estimated that 300, 000 patients are waiting for organ transplantation each year in China, the disparity between supply and demand for organ transplantation is still a conspicuous contradictions.^[8]^ Understanding the beliefs that encourage or dissuade individuals from becoming willing organ donors is therefore of particular importance in China.

While several investigations are conducted gathering the general community views on deceased organ donation in China, they are small set of samples, limited scope area, and a single population.^[9]^ Besides, in these published reports, citizens cognition, attitudes, and willingness towards organ donation are varied, and the listed modifiable factors are also diverse.^[10–12]^ We presented a systematic analysis of the cognition, attitudes, and willingness of the general public on posthumous organ donation in China, through summarizing the published literatures, aimed to form a broad understanding and a more rigorous interpretation of the general public perspectives. This analysis will provide important evidence that directs effective practice efforts and contribute to improve the donation process in China.^[13]^

## 2 Materials and methods

### 2.1 Ethics statement

No ethical approval was needed for this study, because all data were extracted on the basis of a review of published literatures.

### 2.2 Search strategy

Two investigators conducted a systematic review of published peer-reviewed research articles during the period January 1, 1990 to May 31, 2019 by searching the English and Chinese literature databases independently. Databases used in this review included PubMed, China Biology Medicine disc (CBMdisc), VIP Chinese Journal Database (CQVIP), China National Knowledge Infrastructure (CNKI), and Wanfang Data. The search strategy was a combination of Medical Subject Headings and free text terms of (“organ donation” or “organ transplantation”) and (“knowledge” or “attitude” or “willingness”) and (“China” or “Chinese”). A manual search of the reference lists of published articles was also performed. This review was conducted according to the Preferred Reporting Items for Systematic Reviews and Meta-Analyses (PRISMA) statement issued in 2009.^[14]^

### 2.3 Selection criteria

Studies were eligible for inclusion if they met the following criteria:

1. studies were published in Chinese or English language;
2. studies were reported the knowledge and attitudes of organ donation or transplantation and their influencing factors;
3. the studied population was Chinese mainland citizens;
4. study design and sample size were reported.

We excluded studies if they were

1. reviews, comments or presentations;
2. non-peer-reviewed local or government reports;
3. PhD/Master theses;
4. conference abstracts or presentations;
5. studies with incomplete or incorrect data that cannot be extracted;
6. studies with sample size less than 30;
7. studies were done in Hong Kong Special Administrative Region, Macau Special Administrative Region, and Taiwan.

If the same studies were published in both English and Chinese language, the data published in Chinese database were excluded from the review. Two reviewers independently screened the studies, extracted and cross-checked the data. In the event of uncertainty or disagreement, the third reviewer conferred and discussed with each other to reach a consensus.

### 2.4 Quality assessment

The quality of the included literatures was assessed using the critical evaluation criteria of the prevalence or incidence of health problems proposed by Patricia L Loney et al.^[15]^ The criterion evaluates the quality of the studies from a total of 3 aspects and 8 criteria: the validity of the research method, the rationality of the results and the scope of application. Studies with a quality score between 0 and 2 were classified as low quality, those with a score between 3 and 5 as average quality, and those with a score between 6 and 8 as high quality.

### 2.5 Data abstraction

The following information was extracted from all eligible studies: first author, study duration, year of publication, study location, research population, recruitment and sampling method, and sample size. In addition, information about cognition about organ donation and transplantation, the sources of the knowledge about organ donation, attitude and willingness to posthumous organ donation, reasons for refraining from donation, and strategies to improve the willingness to posthumous organ donation was also collected. Since China fully launched DCD in 2015, studies were further categorized into 2 periods of data collection: before 2015 and after 2015, based on the availability of data.

### 2.6 Statistical analysis

Pooled proportions of cognition and attitudes related to organ donation and corresponding 95% confidence intervals (95%CIs) were estimated through the Freeman–Tukey double arcsine transformation by R software (version 3.5.1, Math Soft, Vienna, Austria).^[16]^ Odds ratios (OR) in populations with different demographic characteristics and corresponding 95%CIs were estimated by Stata software (version 12.0, Stata Corp, College Station, Texas, USA). Heterogeneity between different studies was assessed by Cochran's Q-test and the *I*^*2*^ statistic. The heterogeneity was considered to be significant when the *P* value of Cochran's Q-test < 0.1 or *I*^*2*^ > 75%.^[17]^ A fixed-effects model was used when in lack of significant heterogeneity, otherwise a random-effects model was chosen.

## 3 Results

**Figure 1:**
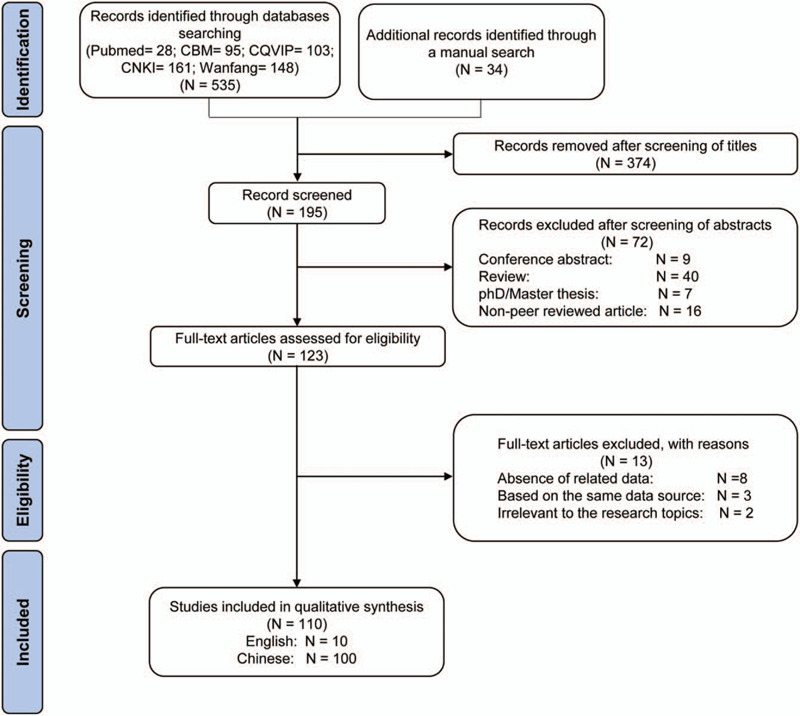
Flow chart summarizing the search strategy and selection procedure.

The initial search criteria identified 535 articles from the 5 electronic databases (PubMed = 28; CBMdisc = 95; CQVIP = 103; CNKI = 161; Wanfang = 148); 34 additional articles were identified through the reference lists of these articles. A total of 374 articles were removed after screening of titles. The abstracts of the remaining 195 articles were screened. Conference abstracts (n = 9), review papers (n = 40), PhD/Master theses (n = 7), and non-peer reviewed articles (n = 16) were excluded. A total of 123 articles were eligible for full-text screening, and 13 were further excluded due to the absence of related data (n = 8), duplicated studies based on the same data source (n = 3), and those irrelevant to the research topics (n = 2) (Fig. 1). The remaining 110 studies (10 in English, 100 in Chinese) were eligible for analysis (see Reference, Supplemental Digital Content 1, which lists references of 110 literatures included in this investigation).

### 3.1 Study characteristics

The publication date of the 110 eligible articles was 1997 to 2019, spanning 23 years. Studies covered 25 provinces of China, involving the most of mainland area (see Table, Supplemental Digital Content 2, which lists the basic information of 110 literatures included in this study). All of the quantitative studies were cross-sectional surveys. Of included articles, 39 were randomly sampled, 16 were stratified, 31 were clustered, 20 belonged to convenience sampling, and 4 belonged to accidental sampling. Groups of studied included whole population (n = 36), college students (n = 28), medical students (n = 21), medical staffs (n = 18), family members of the patient (n = 2), hospitalized patients (n = 1), transplant patients and their families (n = 1), middle school students (n = 1), migrant workers (n = 1) and driving license holders (n = 1). The sampling size ranged from 106 to 12, 205 (median 589, interquartile range 405 to 1051), and the combined sample size was 103, 410. The average quality score of these studies was 5.18 after assessment, among which 0.9%, 57.3%, and 41.8% of the articles were evaluated as low, average and high quality, respectively (see Figure, Supplemental Digital Content 3, which shows the frequency distribution of quality scores of 110 selected articles in this study).

### 3.2 Cognition about organ donation and transplantation

A pooled proportions of 85.6% (77.6–92.1) and 95.0% (92.6–97.0) of participants heard about organ donation and transplantation, respectively (Table 1). The sources of the knowledge about organ donation were primary network (58.3%, 47.2–69.0) and television & radio (55.6%, 49.8–61.4), followed by school-based education (31.5%, 25.4–37.9), publicity materials (26.6%, 19.5–34.4), health professional (25.3%, 21.3–29.4), newspaper & magazine (22.9%, 18.3–27.9), and publicity campaign (18.6%, 9.0–30.7). In addition, the relatives and friends also accounted for 18.4% (12.3–25.4) (see Figure, Supplemental Digital Content 4, which shows the sources of the knowledge about organ donation of Chinese people).

**Table 1:** Chinese citizens’ cognition about organ donation.

	Number of included studies	Sample size	Pooled proportion	95%CI
Heard about organ donation	28	26, 682	85.6	77.6 – 92.1
Heard about organ transplantation	9	12, 576	95.0	92.6 – 97.0

### 3.3 Attitude and willingness to posthumous organ donation

**Figure 2:**
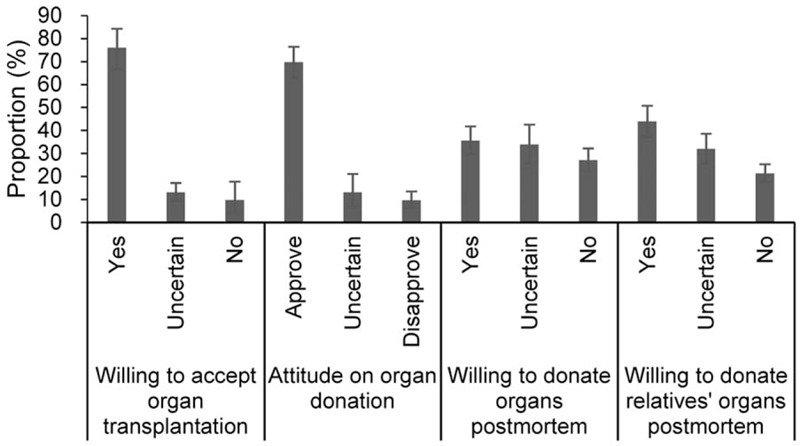
Attitude and willingness to posthumous organ donation. A total of 4 sections are included, including “Willing to accept organ transplantation”, “Attitude on organ donation”, “Willing to donate organs postmortem” and “Willing to donate relatives’ organs postmortem”.

The analysis showed 76.0% (66.6–84.3) of individuals were willing to accept organ transplantation if necessary, and those who were unwilling and uncertain only accounted for 9.8% (4.1–17.8) and 13.1% (9.4–17.2), respectively (Fig. 2). Although 69.7% (62.7–76.4) of participants approved the deceased organ donation, only 35.6% (29.7–41.8) and 43.9% (37.2–50.8) were willing to donate their own and relatives organs postmortem, respectively. Approximately 27.1% (22.4–32.2) and 33.9% (25.7–42.5) of individuals were unwilling and uncertain to donate own organs postmortem, and 21.3% (17.7–25.3) and 32.0% (25.7–38.6) of participants were unwilling and uncertain to donate relatives’ organs postmortem, respectively. The level of education seemed to have a significant impact on individuals’ willingness to donate their own organs postmortem. The participants with university degree or above (47.9%, 38.6–57.4) showed higher willingness than those with high school or less education (42.0%, 36.9–47.2), and OR was 1.22 (1.13–1.31, *P* < .001). A similar finding was also observed for those unwilling to donate own organs postmortem [the pooled proportions with the former and the latter were 47.1% (31.1–63.4) and 50.1% (39.3–60.9), respectively; OR = 0.86 (0.79–0.93), *P* = .001] (see Figure, Supplemental Digital Content 5, which shows the willingness to posthumous organ donation with different education degrees). However, there was no significant difference between the males and females for willingness to donate organs (see Figure, Supplemental Digital Content 6, which shows the willingness to posthumous organ donation with different genders).

### 3.4 Reasons for refraining from donation

**Figure 3:**
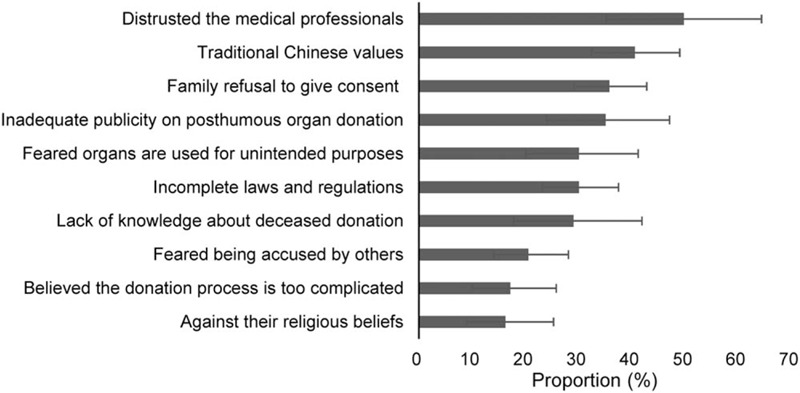
Reasons for refraining from donating organs postmortem.

Figure 3 showed the reasons for refraining from donating organs postmortem. The 5 leading reasons were, “distrusted the medical professionals” (49.8%, 35.2–64.4), “the bodily integrity should be maintained based on traditional Chinese values” (40.6%, 32.4–49.0), “family's disapproval to give consent on deceased donation” (35.8%, 29.1%–42.8), “inadequate publicity on posthumous organ donation” (35.1%, 24.0–47.1), “feared organs are used for unintended purposes” (30.1%, 20.1–41.2), and “believed that the laws and regulations related to organ donation in China were imperfect” (30.1%, 23.2–37.5). In addition, “lack of knowledge about deceased donation” (29.1%, 17.8–41.9), “feared being accused by others” (20.6%, 14.0–28.1), “believed the donation process is too complicated” (17.2%, 10.0–25.8), and “believed that deceased donation is against their religious beliefs” (16.3%, 9.3–25.3) also accounted for certain proportions.

### 3.5 Cognition and willingness to posthumous organ donation by time

**Figure 4:**
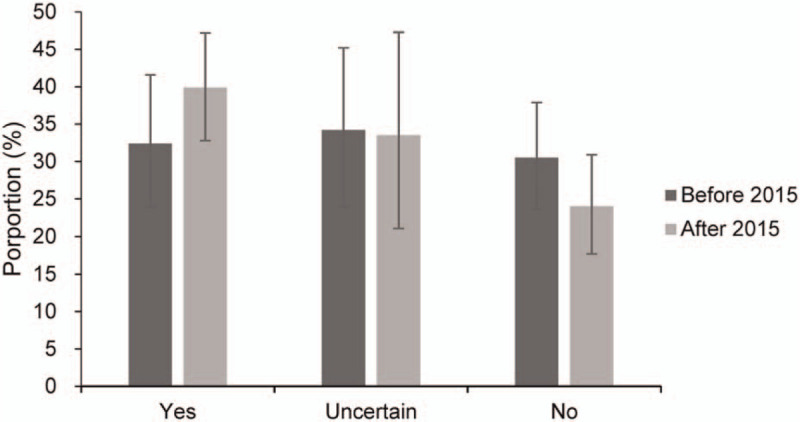
Willingness to posthumous organ donation by time.

Before 2015, the pooled proportion of knowing about organ donation was 84.6% (73.0–93.4), and the figure slightly increased to 86.4% (74.5–95.1) after 2015 (Table 2). Encouragingly, the willingness to posthumous organ donation has a significant improvement since China fully launched DCD in 2015. The proportion of willingness to posthumous organ donation increased to 39.9% (32.8–47.2) after 2015, from 32.4% (23.9–41.6) before 2015. While the proportion of unwilling to donate organs postmortem had decreased from 30.5% (23.6–37.9) before 2015 to 24.0% (17.7–30.9) after 2015 (Fig. 4). Interestingly, the network seemed to play a more significant role in the dissemination of knowledge on organ donation, and the merger proportion increased from 48.1% (29.5–67.1) before 2015, to 66.6% (60.2–72.7) after 2015 (see Figure, Supplemental Digital Content 7, which shows the sources of organ donation knowledge by time).

**Table 2:** The cognition about posthumous organ donation by time.

Period	Number of included studies	Sample Size	Pooled proportion	95%CI
Before 2015	13	14, 428	84.6	73.0 – 93.4
After 2015	15	12, 254	86.4	74.5 – 95.1

### 3.6 Types of organ and tissues willing to donate postmortem

**Figure 5:**
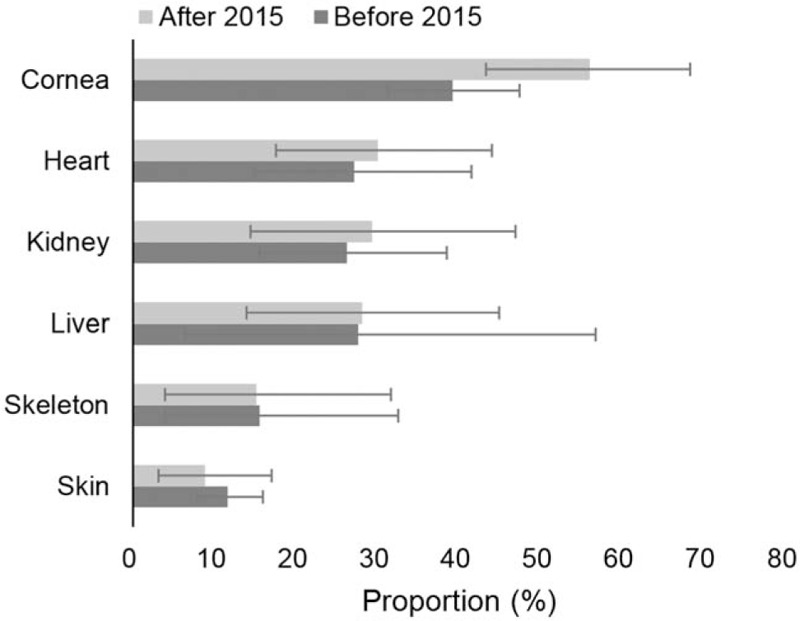
Types of organ and tissues willing to donate postmortem by time.

Among these individuals willing to donate their own organs postmortem, almost half of them [49.8% (39.7–60.0)] were willing to donate cornea. In addition, 29.0% (19.6–39.3), 28.0% (18.5–38.7), 27.9% (16.2–41.4), 21.9% (9.2–38.1) and 16.6% (6.3–30.5) of individuals were willing to donate heart, kidney, liver, lung, and pancreas, respectively. The willingness to posthumous organ donation for cornea, heart, kidney, and liver had a significant improvement since China fully launched DCD in 2015 (Fig. 5). Especially, the proportion of willingness to donate cornea increased to 56.0% (43.3–68.3) after 2015, from 39.2% (31.2–47.4) before 2015. (see Figure, Supplemental Digital Content 8, which shows the types of organ and tissues willing to donate postmortem).

### 3.7 Strategies to improve the willingness to posthumous organ donation

**Figure 6:**
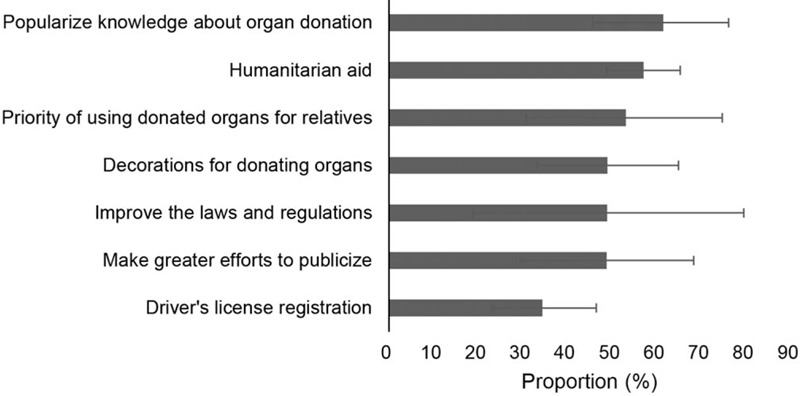
Strategies to improve the willingness to posthumous organ donation.

Figure 6 showed the possible strategies to improve the willingness to posthumous organ donation. The most applauded strategy was, “popularizing knowledge about organ donation” (61.5%, 45.7–76.1). The second most commonly cited strategy was, “humanitarian aid” (57.1%, 48.8–65.3). The following strategies were “priority of using donated organs for relatives” (53.1%, 30.8–74.7), “decorations for donating organs” (49.0%, 33.2–64.9), “improving the laws and regulations” (48.9%, 18.8–79.5), and “making greater efforts to publicize” (48.8%, 29.5–68.3). In addition, 34.4% (23.2–46.5) of participants believed that drivers license registration is an effective approach to improve the willingness to posthumous organ donation.

## 4 Discussion

Due to historical reasons, the international community has widely criticized the source of organs in China in the early 21st century, which threatens the development of organ transplantation.^[1]^ To resolve the issues completely, Chinese government undertook a major reform in this area. An integrated organ donation system including regulations and policies, an organizational structure, operational guidelines, organ procurement organizations, registration of donors and recipients, as well as an organ allocation system have been developed, which ensures fairness, impartiality, and transparency throughout the process of organ donation.^[18–20]^ In this paper, a systematic analysis was used to identify the cognition, attitudes and willingness towards deceased organ donation for Chinese population. In generally, the development of organ donation has a close relationship with the improvement and progress of national policies. After years of continuous efforts, the willingness to posthumous organ donation has a significant improvement among Chinese general community; however, the favorable attitudes and willingness toward organ donation still remained at a low level.

The public has relatively less access to organ donation activities in China and is skeptical of the recognition and prevalence of organ donation.^[21]^ Mistrust of the medical profession was the greatest impediment for Chinese citizens willing to donate organs postmortem.^[22]^ On the one hand, public were afraid that organs would be dismembered unethically and body would be disfigured. Some, for instance, feared that doctors go beyond an individuals terms of consent to obtain additional organs, or procured organs rudely.^[23]^ On the other hand, individuals feared that medical staff would deliberately remove a patients organs before the patient had died, or life-saving medical care would be withheld excessively so that patients could become eligible for organ donation.^[24]^ Moreover, a considerable proportion of public believed that the laws and regulations related to organ donation in China were incomplete. They feared that organs could be used for unintended purposes, such as illicit trading of organs or unauthorized medical experiments.^[25]^ In recent years, newspapers, TV news, programs, and the internet have covered many cases of organ donation and transplantation. Overwhelming majority of propagation, nevertheless, was around the theme of altruism spirit for saving lives for others, rather than the knowledge on organ donation and transplantation.^[26]^ These common worries and distrust highlights the importance to strengthen targeted publicity about regulations and policies, operational guidelines, and allocation system of organ donation.

Due to the impact of Chinese traditional beliefs, many individuals believed that the bodily integrity should be maintained to safeguard progression into the afterlife.^[27]^ Especially, considerable individuals rejected organ donation because some parts of the body, eyes, and heart for instance, were thought to hold particular significance. In some ethnic minorities, donation needed to be approved by the ancestor or Shaikh, so that the remaining family did not lose ancestral protection in the future. Some participants even held the superstitious beliefs that discussing death or signing a donor card would lead one's own death. Thus, it may need the sustained efforts in all quarters of our society to get rid of the shackles of traditional concept. In China, family consent to donation is an essential step in organ procurement; organs would not be retrieved from a dead patient against the wishes of his or her family.^[28]^ Family disapproved to give consent remained a major barrier for donating organs. Strategies need to be adopted to help families eliminate misunderstandings, correct traditional concepts, distinguish death criteria, understand the donation process and background, to cultivate trust in the donation process, solve the insecurity in the decision-making process.

The results showed that about 85.6% and 95.0% of participants knew about organ donation and transplantation, respectively, indicated Chinese citizens has a degree of cognition about this field. Whereas several studies reported that the overall knowledge rates for Chinese citizens remains at a low level, especially about the sector where to donate, procedure of donation, and brain death were seriously lacking.^[21]^ In addition, a disturbing number of individuals supposed that lack of knowledge (29.1%) and inadequate publicity (35.1%) on posthumous organ donation hindering public become an organ donor. Therefore, the future donation campaigns and education programs in China should be held as long-standing campaigns, in case of their impact on community attitudes decreased over time. In spite of support for donation in principle in the general public, this is not always reflected in the actual behavior of donation, only 35.6% and 43.9% of individuals were willing to donate their own and relatives’ organs postmortem, respectively. This indicated that there is far from cognition to the actually organ donations completed. It is necessary to develop and implement effective educational campaigns to increase awareness, dispel myths, and modify attitudes about donation among the public. Of note, approximately a quarter of individuals were uncertainty for donating organs postmortem, which indicated that although a part of Chinese citizens had not been prepared for donation, but they were not against donation. Thus, efforts need to be made to motivate these potential donors to be bona fide donors.

The public suggested several strategies to improve the willingness to posthumous organ donation. From the feedback of the public, popularizing knowledge about organ donation was the most applauded, which indicated that the public believes the popularization of organ donation-related knowledge, detailed donation process, donation policy and so on can play important roles. At present, many strategies have been implemented in China. For instance, the Basic Principles of Chinese Human Organ Distribution and Sharing and the Core Policy, promulgated in July 2018,^[29]^ which stipulates the regimen for get an organ preferentially. In addition, an increasing number of memorial cemeteries were also built, and all organ donors were certificatoried.^[30]^ These strategies played an important role in improving the willingness to posthumous organ donation. However, the promotion strategy is still not perfect in China, the laws and regulations should be improved, and the publicity needs to be strengthened in the further.

## 5 Limitation

There are some limitations in this study. We set out to synthesize general public attitudes to organ donation, while certain groups, such as college students, medical students, and medical staffs were overrepresented in the reviewed studies. Consequently, the results of this review are perhaps skewed towards the positive results as many of these specific populations have a high degree of education than general public. Although this analysis provides several important perspectives on organ donation for Chinese population, many of these views are only shaped the outline and without delving into details given the limited primary literatures. Several key thematic categories that impacted on individuals willingness to become an organ donor deserves further study rooted in native culture.

## 6 Conclusions

After years of continuous reformation, the willingness toward posthumous organ donation has a significant improvement among Chinese general public since 2015, however, the favorable attitudes and willingness toward organ donation still remained at a relatively low level. Future work should be focused on trumpeting the reform achievements and providing comprehensive information about deceased organ donation, to reassure public, dispel misperceptions, and improve the willingness toward organ donation.

## Author contributions

Xiaoshan Li, Rong Gao and Jingyu Chen conceived and designed the study. Di Hu, Gongtao Qian, Dong Wei, Jianmei Zhou, Lihua Zhang, and Weiwei Xu prepared the data. Xiaoshan Li, Junyan Miao and Di Hu analyzed the data. Xiaoshan Li, Junyan Miao and Chunxiao Hu wrote the paper. All authors read and approved the final manuscript.

## Supplementary Material

Supplemental Digital Content

## Supplementary Material

Supplemental Digital Content

## Supplementary Material

Supplemental Digital Content

## Supplementary Material

Supplemental Digital Content

## Supplementary Material

Supplemental Digital Content

## Supplementary Material

Supplemental Digital Content

## Supplementary Material

Supplemental Digital Content

## Supplementary Material

Supplemental Digital Content
