# Supplementary material for: The general public new views on deceased organ donation in China
Source: Medicine (Baltimore). 2020 Dec 11;99(50):e23438. doi: 10.1097/MD.0000000000023438 (PMC7738062; doi:10.1097/MD.0000000000023438)
Supplement: Supplemental Digital Content [file medi-99-e23438-s001.docx]

Supplemental Digital Content (SDC 1)

**The general public new views on** **deceased organ donation in China**

*Xiaoshan Li, PhD, Junyan Miao, BM, Rong Gao, PhD*

**SDC 1. References of the 110 literatures**.

1. Tian H, Wang W, Yin H, et al. The public view of organ donation in China: analysis of questionnaire results (in Chinese). Chin J Organ Transplant 2011; 32: 726-729.
2. Liu YL, Lei H, Qiu FZ. Investigation of understanding and willingness of organ transplantation in young people in Beijing, Shanghai, Wuhan (in Chinese). Natl Med J China 1997; 77:22-27.
3. Liu J, Ouyang YN, Wu C, et al. Cognition in human organ transplantation: comparative study between medical and non-medical college students in Beijing (in Chinese). Organ Transplantation 2014; 5: 103-106.
4. Huang N, Zhou YM, Wu CX, et al. Investigation and analysis on the cognition and influencing factors of body donation of students in North Sichuan Medical College (in Chinese). Investigation and study 2017; 58.
5. Wang L, Tian R, Yang MY. A study of College Students' cognition and attitude towards organ donation (in Chinese). ISSN2095-9923, 83-84.
6. Sun JP, Gao YP, Wang LH. Cognition to organ transplantation and organ donation in college students (in Chinese). Medicine and Society 2005; 18: 1-4.
7. Tang M, He Y. Medical students’ Cognition and Attitudes towards body donation (in Chinese). Journal of Yangzhou University (Higher Education Study Edition) 2017; 21: 51-55.
8. Wang CF, Du Z, Dai GA. Investigation and analysis of College Students' donation cognition and related course construction (in Chinese). China Higher Medical Education 2013; 4: 47-48.
9. Sun HS, Guo HX, Jia LH. Analysis on attitude toward organ donation and its effect factors among college students (in Chinese). J Henan Univ Sci Tech (Med Sci) 2006; 24: 149-150.
10. Wu HY. A survey of attitude and cognition of human organ transplantation among college students (in Chinese). Medicine and Philosophy (Humanistic &Social Medicine Edition) 2008; 29: 18-27.
11. Sun XY, Fu JZ, Xia JZ, et al. A survey of the attitude and related cognition of organ transplantation and donation between university teachers and students (in Chinese). Health Way 2016; 15: 235.
12. Jiao YL, Gao L, Jin YL. Investigation of the status of the experience of persuading to donate and the willingness toward organ donation for ICU nurses (in Chinese). Chin J Mod Nurs 2013; 19: 1745-1748.
13. Liu H, Zhang Y, Xu LZ. Survey on knowledge, attitude and practice towards potential organ donors coordinating donation in clinical nurses (in Chinese). Chin J Mod Nurs 2015; 21: 1389-1392.
14. Zhong J, Li SY, Zhu SG, et al. Survey on general public’s attitude to organs donations (in Chinese). Journal of Baotou Medical College 2016; 32: 8-10.
15. Luo WD, Jin WZ, Zou H, et al. The investigation of the body donation will and influence factors of people in Yunnan province (in Chinese). Chinese Medical Ethics 2015; 28: 36-38.
16. Guo ZX, Wei SW, Zhang CY. Willingness and reasons of non-medical person for organ donation (in Chinese). Chin J Mod Nurs 2014; 20: 1506-1509.
17. Yan JL, Yuan HY. Investigation and analysis on citizens’ cognition of living organ donation (in Chinese). Chinese Medical Ethics 2017; 30: 787-791.
18. Li Y. The diagnoses of populace attitude to transplant of vital organs of living body (in Chinese). Medicine and Philosophy (Clinical Decision Making Forum Edition) 2007; 28: 47-56.
19. Zhu SJ, Zhang J, Cui YJ, et al. Investigation and analysis of public cognition and willingness to organ donation (in Chinese). J Med Theor & Prac 2017; 30: 1556-1558.
20. Zeng CY, Zhu YZ. Current situation and the influencing factors of organ donation cognition in university students in Wenzhou Chashan District (in Chinese). Chinese Medical Ethics 2014; 27:869-872.
21. Lin JY, Pang ZM. A survey of cognition and attitude of organ donation among college students in Guangzhou University Town (in Chinese). Medical frontier 2013; 38: 94-95.
22. Zhang FF, Xia F, Pang ZM. A survey of cognition and attitude towards organ donation among medical college students in Guangzhou (in Chinese). Chinese journal of ethno medicine and ethno pharmacy 2013; 6: 59-61.
23. Zhou JH, Li MY, Li HY, et al. Survey and analysis of the factors affecting civil will towards donating cadaver in Guangzhou (in Chinese). Chinese Medical Ethics 2004; 17: 40-48.
24. Li Y. A survey of residents' cognition and attitude towards living organ transplantation in Henan Province (in Chinese). Chin J Public Health 2007; 23: 758.
25. Hu YJ, Shen MD, Wang Y, et al. Cognition on cadaver donation and its influencing factors among residents in Henan province (in Chinese). Chin J Public Health 2017; 33: 1148-1151.
26. Chen XQ, Zhang B, Tang LX, et al. A study on the knowledge, attitude and influence factors of the donation of remains in Huzhou (in Chinese). Health Vocational Education 2010; 28: 103-104.
27. Zhang J, Xie LF, Song YX, et al. Investigation on cognition, willingness and curriculum needs of nursing undergraduates on organ donation and transplantation (in Chinese). Chinese Nursing Research 2016; 30: 3779-3782.
28. Dang W, Jiang H. Investigation and analysis on current status of organ donation willingness and death attitudes of medical students majoring in nursing (in Chinese). Organ Transplantation 2017; 8: 460-464.
29. Wang YL. Study of the cognition and willingness to organ donation based on SPSS (in Chinese). Yinshan Academic Journal 2017; 31: 94-96.
30. Tang R, Zhu JM. The study of organ donation willingness based on single factor method (in Chinese). Journal of Jinzhou Medical University (Social Science Edition) 2017; 15: 15-17.
31. Liu DD, Zhu JM, Huang TT. Based on logistic regression and factor analysis of organ donation willingness research (in Chinese). Journal of Qiqihar University (Natural Science Edition) 2017; 33: 76-82.
32. Wang TX, Song LJ, Wang T, et al. Cognition of and attitude towards organ donation among urban residents in Ji'nan city (in Chinese). Chin J Public Health 2014; 30: 645-647.
33. Zhang HC, Zheng JN, Liu WH, et al. Investigation and strategic analysis of public attitudes and willingness towards organ donation in Jiangsu (in Chinese). Aata Universitatis Medicinalis Nanjing (Social Sciences) 2015; 05: 339-342.
34. Zheng YN, Gong X, Liu M. Investigation and analysis of medical students’ attitude towards remains donation and the influencing factors in a medical college in Jiangxi (in Chinese). Chinese Journal of Anatomy 2017; 40: 772-773.
35. Li D, Lu JH. Investigation on cognition of body donation and analysis on its influencing factors among clinical medicine students (in Chinese). Occup and Health 2016; 32: 1533-1535.
36. Wang YJ, Li C, Zhang R, et al. Investigation of influential factors for aspiration of organ donation (in Chinese). Organ Transplantation 2013; 4: 75-78.
37. Mo YW, Zhang WH, Mo HM. A study on cognition and willingness of organ transplantation and donation (in Chinese). Modern Medicine and Health Research 2017; 1: 141.
38. Chen XW, Dai WY, Li CJ. The influence of life education on the willingness of medical students to donate organs (in Chinese). China Higher Medical Education 2016; 9: 27-28.
39. Liu C, Liu SQ, Liu BL. The promoting effect of moral education toward cadaveric organ donation among medical students (in Chinese). China Higher Medical Education 2014; 11: 45-46.
40. Wang N, Yan ZX, Wang XM, et al. An investigation into the attitudes towards organ transplantation and willingness of organ donation in college students-based on the questionnaire in Sichuan province (in Chinese). Journal of Chengdu Medical College 2016; 11: 250-253.
41. Chen JZ, Liu XM, Ni YH. An investigation and analysis of Suzhou Vocational College Students' awareness and willingness to donate human organs (in Chinese). Examination Weekly 2015; 90: 150-151.
42. Lei J, Zhao JW, Bai F, et al. The investigation of cognitive status about organ donation of teachers and students in colleges of Suzhou (in Chinese). Chinese Journal of Social Medicine 2017; 34: 159-161.
43. Tan YL, Xie WF, Chen L, et al. A survey on the recognition of Chinese ICU medical staff to heart dead organ donation (in Chinese). China Medical Engineering 2015; 23: 112-113.
44. Yang Y, Huang H, Qiu HZ. Study on the willingness and influence factors of organ donation after death of citizens in China (in Chinese). Chinese Hospitals 2014; 18: 18-19.
45. Wang L, Dong Q, Li YP, et al. A survey on the cognition and willingness of Chinese medical workers to organ transplantation (in Chinese). Medicine and Philosophy 2202; 23: 62-63.
46. Zhang C, Zhu XR, Chen SW, et al. An investigation and analysis of the willingness and influencing factors to donate remains (organs) of Wuhu citizens (in Chinese). J Med Theor & Prac 2016; 29: 1383-1384.
47. Shen XK, Dai JB, Song HN, et al. Investigation on the knowledge, the attitude and the behavior of Wuhan citizens to remains donation (in Chinese). Soft Science of Health 2004; 18: 194-197.
48. Luo WD, Wan ZY, Zou H, et al. The investigation on the will and its influence factors of the Yi, Dai and Han people in Yunnan province about body donation (in Chinese). Journal of Kunming Medical University 2013; 12: 19-25.
49. Wang CJ, Chen X, Lu X, et al. Investigation and analysis of awareness and willingness of medical staff to participation in organ donation (in Chinese). Ability and Wisdom 2016; 234-235.
50. Wang J, Zhang ZM. An empirical study of medical staffs' attitude toward organ transplantation ethics and collect donations (in Chinese). Chinese Health Service Management 2013; 3: 232-235.
51. Xi H. The investigation and analysis of medical students’ viewpoint on organ replant (in Chinese). Chinese Medical Ethics 2001; 6: 12-16.
52. Lu YK, Zeng RH, Zhu TT. Comparison between students from a medical university and a non-medical university on the recognition of body donation and the attitude towards it (in Chinese). Acta Universitatis Medicinalis Nanjing (Social Sciences) 2016; 2: 125-129.
53. Ma GH, Liu XH, Liu ZM, et al. A survey of medical students' cognition and attitude towards organ donation (in Chinese). Harbin Medical Journal 2017; 37: 571-572.
54. Hu DM, Huang H. The attitudes of medical students regarding to organ donation compensation (in Chinese). Chin J Organ Transplant 2015; 36: 176-179.
55. Li YL, Song AQ, Li N, et al. The research on the medical students’ willing of body donation (in Chinese). J Jining Med Univ 2015; 38: 145-148.
56. Liu C, Liu SQ, Liu BL, et al. Freshman’s attitude on cadaveric organ donation and its influencing factors (in Chinese). Chinese Medical Ethics 2013; 26: 559-561.
57. Tang X, Xiao X, Cao DL, et al. Comparing the Difference between the Medical Student and Non-medical Student on Postmortem Donation (in Chinese). Medicine and Philosophy 2012; 33: 25-26.
58. Jin C, Wei LQ, Wu JL, et al. A Survey on the Awareness and Influencing Factors of Body Donation among Medical College Students (in Chinese). Chinese Medical Ethics 2011; 24: 760-761.
59. Liu BL, Shang B, Liu SQ, et al. The attitude of hospital personnel toward living organ donation and analysis of its impact factors (in Chinese). Journal of China Medical University 2011; 40: 428-430.
60. Li C, Zhang R, Wang YJ, et al. Questionnaire analysis of attitude towards organ transplantation and donation in inpatients (in Chinese). Chin J Bases Clin General Surg 2012; 19: 478-485.
61. Tu DD, Xu Z, Liu X. A comparative study on cognition and willingness of organ donation between medical students and non-medical students——Take Chengdu University of traditional Chinese medicine and Southwest University of Finance and economics as examples (in Chinese). Special health 2017; 24: 47.
62. Xian WW, Wang YR, Chen JL, et al. The attitude of college students to organ donation (in Chinese). Magnificent Writing 2014; 23: 158.
63. Tao J, Lu SF, Sun YQ, et al. A study on the cognition and attitude of medical students to organ donation (in Chinese). Health Vocational Education 2015; 33: 120-121.
64. Du CC, Li N, Zhou W, et al. Investigation on attitude and cognition of medical students to organ donation (in Chinese). Medical Information 2013; 26: 411.
65. Zhang XW, Li GY, Li Z, et al. Investigation of medical students’ attitude and cognition to human organ transplantation and donation (in Chinese). J Clin Rehabil Tis Eng Res 2012; 16:10067-10070.
66. You YW, Hao L, Chen XM, et al. A questionnaire analysis of the factors affecting the civil attitudes towards cadaver donation in Zhengzhou (in Chinese). Chinese Medical Ethics 2009; 22: 98-100.
67. Si J, Huang W, Gu Y, et al. A study of factors influencing family decision of Chinese donation after citizen's death (in Chinese). Chin J Transplant (Electronic Edition) 2015; 9: 174-177.
68. Ma LL, Zhao L, Yu LX, et al. A questionnaire survey on cognition and intention towards organ donation in driving license holders of Mainland China (in Chinese). Chin J Urol 2014; 35: 10-14.
69. Lei L, Zhang HL, Dong H, et al. Medical and non-medical college student perceptions of organ donation in Chongqing: a cross-sectional study (in Chinese). Journal of Nursing Science 2017; 3: 96-99.
70. Mou SL, Wang L Mou SY, et al. Investigation on the cognition about organ donation of ICU medical staff in 3A hospital in Chongqing (in Chinese). Chin J Prac Nurs 2016; 32: 2810-2813.
71. Yang CH, Chen XX, Chen L, et al. Intensive care unit survey of cognition status of donation after brain death (in Chinese). Natl Med J China 2014; 94: 1570-1572.
72. Pan XM, Liu LJ, Xiang HL, et al. Current attitudes toward organ donation after cardiac death in northwest China. Chinese Medical Journal 2014; 127: 835-838.
73. Zhang QX, Xie JF, Zhou JD, et al. Impact factors and attitudes toward organ donation among transplantation patients and their caregivers in China. Transplantation Proceedings 2017; 49: 1975-1981.
74. Zhang H, Zheng J, Liu W, et al. Investigation and strategic analysis of public willingness and attitudes toward organ donation in east China. Transplantation Proceedings 2015; 47: 2419-2424.
75. Zhang L, Li Y, Zhou J, et al. Knowledge and willingness toward living organ donation: A survey of three universities in Changsha, Hunan province, China. Transplantation Proceedings 2007; 39: 1303–1309.
76. Hu DM, Huang H. Knowledge, attitudes, and willingness toward organ donation among health professionals in China. Transplantation 2015; 99: 1379-1385.
77. Luo AJ, Xie WZ, Luo JJ, et al. Public perception of cadaver organ donation in Hunan province, China. Transplantation Proceedings; 2016: 48, 2571-2576.
78. Yan J, Wang Y, Shao J, et al. Attitudes toward living organ donation and willingness to be a living organ donor among Chinese citizens in 3 cities. Transplantation Proceedings 2018; 50: 3065-3070.
79. Zhang JX, Chen XS, Yang YW, et al. Attitude toward and willingness of organ donation among 330 college students (in Chinese). Journal of Nursing (China) 2018; 25: 21-24.
80. Wang R, Huang F, Liu JQ, et al. Investigation of knowledge’ attitude and willingness on organ donation of undergraduate students from different disciplines (in Chinese). Journal of Nursing Administration 2013; 13: 570-572.
81. Wang LP, Chen LL, Chen MQ, et al. Research on the attitude，knowledge and intention of organs donation in Dongguan colleges’ students (in Chinese). Nursing Prac Res 2018; 15: 12-14.
82. Liang YQ, Chen W, Zhang Z, et al. Investigation on cognition, attitude and willingness toward organ donation among students of a university in Guangzhou city (in Chinese). Medicine and Society 2017; 30: 48-61.
83. Gao Q, Huang XH, Fang Q, et al. An investigation on knowledge, attitude and willingness of human organ donation among undergraduate students in Hangzhou city (in Chinese). Zheijimg Prev Med 2015; 27: 1003-1007.
84. Zhan J, Li MR, Liu Q, et al. A survey of cognition and willingness of medical staff to organ donation in Xianger, Meishan (in Chinese). Health Care Guide 2018; 41: 365.
85. Shan L, Jiang XM, Lv XY, et al. Willingness of organ donation and its influencing factors in Xianger, Meishan (in Chinese). Health Care Guide 2018; 41: 380.
86. Ji BX. Investigation on the knowledge and attitudes of organ donation of ICU medical staff in a 3A hospital (in Chinese). Med Edu Mgt 2018; 4: 338-341.
87. Huang F, Zhou JL, Liu JQ, et al. Knowledge, attitude and willingness of organ donation among rural residents in Southwest of Hunan province (in Chinese). Chin J Public Health 2013; 29: 417-419.
88. Du YY, Sun L, Lv Y. Cognition, attitude and willingness of university students towards organ donation in Tianjin city (in Chinese). Medicine and Society 2018; 31:59-62.
89. Liu BJ, Li N. Investigation of willingness of medical stuff towards organ donation and its influencing factors (in Chinese). Beijing Medical Journal 2017; 39: 1194-1200.
90. Luo XY, Wu HZ, Liu Y, et al. Investigation on knowledge, attitude and willingness of organ donation among medical students, northern Guangdong province (in Chinese). Modern Preventive Medicine 2016; 43: 3164-3168.
91. Xiao DL, Wan YP, Huang F, et al. Knowledge, willingness of and attitude toward organ donation among higher vocational school students (in Chinese). Journal of Nursing (China) 2015; 22: 32-35.
92. Dong H, Xu X, Zhang JS, et al. Survey of current status and influencing factors analysis of attitude and willingness of ICU nurses towards organ donation in Chongqing (in Chinese). Organ Transplantation 2019; 10: 61-66.
93. Xie JF, Wang CY, He GP, et al. Attitude and impact factors toward organ transplantation and donation among transplantation nurses in China. Transplantation Proceedings 2017; 49: 1226-1231.
94. Zhang H, Jiao CY, Li MY. Survey on knowledge，attitudes, willingness of health workers and patients with their families towards organ donation (in Chinese). Acta Universitatis Medicinalis Nanjing (Social Sciences) 2018; 6: 438-442.
95. Gen ZX, Niu TF, Gao SC, et al. Investigation of influencing factors of residents' willingness to donate organs in Yantai City and conception of novel propaganda mode (in Chinese). Organ Transplantation 2018; 9: 445-450.
96. Zhou XN, Zhang YQ, Xia T, et al. Analysis of status quo and influencing factors of knowledge, attitude and behavior of nurses on organ donation coordination (in Chinese). Chinese Nursing Research 2016; 30: 3622-3626.
97. Jiao YL, Gao L, Jin YH. Survey of status quo of organ donation knowledge and attitudes of nurses in ICU (in Chinese). Chinese Nursing Research 2013; 27: 2100-2102.
98. Liu JQ, Deng SS, Leng B, et al. Knowledge, attitude and willingness of organ donation of College Students (in Chinese). Journal of Qilu Nursing 2012; 18: 61-63.
99. Zhou JL, Zhang YJ, Liu ZH, et al. Investigation on the status quo of baccalaureate nursing students on the knowledge , attitude and willing toward organ donation (in Chinese). Chinese Nursing Research 2011; 25: 2088-2089.
100. Wu HY. Investigation of undergraduates’ attitude and cognition on human organ donation (in Chinese). Journal of Clinical Rehabilitative Tissue Engineering Research 2008; 12: 6125-6129.
101. Liang SM, Liang YT, Ma MG, et al. Investigation and analysis of the recognition and willingness of organ donation by residents in Nanning City (in Chinese). Medical Information 2019; 32: 129-135.
102. Hua J, Ren L, Chen D, et al. A survey of cognition and attitude towards organ donation among medical students in a Medical College (in Chinese). Journal of Clinic Nursing's Practicality 2019; 4: 80-84.
103. Jia LN, Xiao QG, Yang QL, et al. A study of factors influencing family decision of donation after citizen’s death in northwest China (in Chinese). Chinese Medical Ethics 2019; 32: 474-478.
104. Li Y. A study of population's knowledge and readiness about organ transplant and donation and corresponding suggestions (in Chinese). Journal of Xichang University (Natural Science Edition) 2019; 33: 96-100.
105. Zeng H, Lv YY, Liu RH, et al. Investigations on the willingness of organ donation and its impact factors among middle school students (in Chinese). Prac J Organ Transplant (Electronic Version) 2018; 6: 453-457.
106. He H, Cheng LJ, Ye P, et al. Willingness of medical workers to transplant and donate organs in a hospital in Chengdu (in Chinese). World Latest Medicne Information (Electronic Version) 2018; 18: 190-191.
107. Dai AP, Li HO, Zeng XH, et al. Study on effect organ donation intervention for medical students (in Chinese). Chinese Nursing Research 2015; 29: 1378-1380.
108. Zhang WY, Liu YY, Shi XJ, et al. Public knowledge and attitude toward organ donation in Tianjin, China. Transplantation 2017; 101: 68-69.
109. Wu Z, Gao LL, Hu AL, et al. Willingness to communicate about organ donation among migrant workers in mainland China: a cross--sectional questionnaire survey. International Medicine & Health Guidance News 2017; 23: 3477-3483.
110. Xie QJ, He XL, Zhang ZL, et al. A survey of the willingness to donate human organ remains in Bijie City (in Chinese). Journal of Medical Aesthetics and Cosmetology 2018; 27: 19-20.
