# Supplementary material for: The general public new views on deceased organ donation in China
Source: Medicine (Baltimore). 2020 Dec 11;99(50):e23438. doi: 10.1097/MD.0000000000023438 (PMC7738062; doi:10.1097/MD.0000000000023438)
Supplement: Supplemental Digital Content [file medi-99-e23438-s002.docx]

Supplemental Digital Content (SDC 2)

**The general public new views on** **deceased organ donation in China**

*Xiaoshan Li, PhD, Junyan Miao, BM, Rong Gao, PhD*

**SDC 2. The basic information of the literatures.**

| Serial number | First author | Study duration | Publication time | Province/City | Population | Sample  size | Quality  score |
| --- | --- | --- | --- | --- | --- | --- | --- |
|  |  |  |  |  |  |  |  |
| 1 | Tian H |  | 2011 | Beijing | Whole population | 2, 930 | 6 |
| 2 | Liu YL |  | 1997 | Beijing, Shanghai, Wuhan | Whole population | 3, 012 | 6 |
| 3 | Liu J |  | 2014 | Beijing | College students | 882 | 6 |
| 4 | Huang N |  | 2017 | Sichuan | Medical students | 552 | 3 |
| 5 | Wang L |  | 2017 | Xian | College students | 456 | 3 |
| 6 | Sun JP | 2002-2003 | 2005 | Hebei | College students | 916 | 6 |
| 7 | Tang M |  | 2017 | Nanjing | Medical students | 298 | 5 |
| 8 | Wang CF |  | 2013 | Shandong | College students | 522 | 4 |
| 9 | Sun HS |  | 2006 | Henan | College students | 462 | 5 |
| 10 | Wu HY |  | 2008 | Sichuan, Jiangsu | College students | 544 | 3 |
| 11 | Sun XY |  | 2016 | Fujian | College teachers and students | 491 | 6 |
| 12 | Jiao YL | 2012 | 2013 | Beijing | ICU medical staff | 194 | 4 |
| 13 | Liu H | 2013 | 2015 |  | Nurses | 353 | 5 |
| 14 | Zhong J |  | 2016 | Anhui | Whole population | 1, 038 | 5 |
| 15 | Luo WD |  | 2015 | Yunnan | Whole population | 500 | 6 |
| 16 | Guo ZX | 2013 | 2014 | Gansu | Non-medical personnel | 504 | 6 |
| 17 | Yan JL | 2016 | 2017 | Shanghai | Whole population | 1, 063 | 5 |
| 18 | Li Y | 2005 | 2007 | Henan | Whole population | 565 | 6 |
| 19 | Zhu SJ | 2016 | 2017 | Zhejiang | Whole population | 1, 326 | 5 |
| 20 | Zeng CY |  | 2014 | Zhejiang | College students | 279 | 4 |
| 21 | Lin JY | 2013 | 2013 | Guangzhou | College students | 485 | 4 |
| 22 | Zhang FF |  | 2014 | Guangdong | Medical students | 226 | 6 |
| 23 | Zhou JH | 2002-2003 | 2004 | Guangdong | Whole population | 753 | 4 |
| 24 | Li Y | 2005 | 2007 | Henan | Whole population | 565 | 5 |
| 25 | Hu YJ | 2015-2016 | 2017 | Henan | Whole population | 1, 108 | 7 |
| 26 | Chen XQ |  | 2010 | Zhejiang | Whole population | 204 | 5 |
| 27 | Zhang J | 2015 | 2016 | Anhui | Medical students | 621 | 6 |
| 28 | Dang W | 2017 | 2017 | Tianjin | Medical students | 409 | 6 |
| 29 | Wang YL |  | 2017 |  | Whole population | 1, 350 | 3 |
| 30 | Tang R |  | 2017 | Zhejiang | Whole population | 1, 350 | 6 |
| 31 | Liu DD |  | 2017 | Zhejiang | Whole population | 1, 350 | 6 |
| 32 | Wang TX | 2012 | 2014 | Shandong | Whole population | 400 | 5 |
| 33 | Zhang HC | 2014-2015 | 2015 | Jiangsu | Whole population | 742 | 6 |
| 34 | Zhen YN | 2016 | 2017 | Jiangxi | Medical students | 920 | 5 |
| 35 | Li D |  | 2016 | Jiangsu | Medical students | 204 | 6 |
| 36 | Wang YJ | 2009-2010 | 2013 | Kunming | Whole population | 973 | 5 |
| 37 | Mo YW |  | 2017 | Zhejiang | Whole population | 1, 350 | 4 |
| 38 | Chen XW |  | 2016 | Zhejiang | Medical students | 338 | 5 |
| 39 | Liu C |  | 2014 | Liaoning | Medical students | 546 | 5 |
| 40 | Wang N |  | 2016 | Sichuan | College students | 2, 069 | 7 |
| 41 | Chen JZ | 2012-2013 | 2015 | Jiangsu | College students | 1, 533 | 5 |
| 42 | Lei J |  | 2017 | Jiangsu | College teachers and students | 475 | 5 |
| 43 | Tan YL | 2014 | 2015 | Nationwide | ICU medical staff | 1, 079 | 5 |
| 44 | Yang Y | 2013 | 2014 | Beijing, Shanghai, Chongqing, Guangzhou | Whole population | 863 | 5 |
| 45 | Wang L |  | 2002 | Nationwide | Medical staff | 586 | 4 |
| 46 | Zhang C | 2015 | 2016 | Anhui | Whole population | 534 | 5 |
| 47 | Shen XK |  | 2004 | Hubei | Whole population | 923 | 6 |
| 48 | Luo WD |  | 2013 | Yunnan | Whole population | 1, 076 | 6 |
| 49 | Wang CJ |  | 2016 | Anhui | Medical staff | 618 | 5 |
| 50 | Wang J | 2011-2012 | 2013 | Jiangsu | Medical staff | 887 | 4 |
| 51 | Xi H |  | 2001 | Liaoning | Medical students | 200 | 3 |
| 52 | Lu YK |  | 2016 | Jiangsu | College students | 303 | 5 |
| 53 | Ma GH |  | 2017 | Ningxia | Medical students | 860 | 4 |
| 54 | Hu DM | 2013-2014 | 2015 |  | Medical students | 592 | 6 |
| 55 | Li YL |  | 2015 |  | Medical students | 1, 323 | 6 |
| 56 | Liu C |  | 2013 | Liaoning | Medical students | 564 | 6 |
| 57 | Tang X | 2011 | 2012 | Liaoning | College students | 2, 402 | 6 |
| 58 | Jin C | 2011 | 2011 | Inner mongolia | Medical students | 412 | 4 |
| 59 | Liu BL | 2008 | 2011 |  | Medical staff、citizens | 750 | 5 |
| 60 | Li C | 2010-2011 | 2012 | Yunnan | Hospitalized patients | 906 | 6 |
| 61 | Tu DD |  | 2017 | Sichuan | College students | 161 | 3 |
| 62 | Xian WW |  | 2014 | Heilongjiang | College students | 300 | 2 |
| 63 | Tan J |  | 2015 | Zhejiang | Medical students | 208 | 4 |
| 64 | Du CC | 2013 | 2013 | Sichuan | Medical students | 364 | 5 |
| 65 | Zhang XW | 2011 | 2012 | Shandong | Medical students | 381 | 5 |
| 66 | You YW |  | 2009 | Henan | Whole population | 724 | 6 |
| 67 | Si J | 2010-2015 | 2015 | Hubei | ICU family members of the patients | 1, 436 | 7 |
| 68 | Ma LL | 2012 | 2014 | Nationwide | Driving license holders | 12, 205 | 6 |
| 69 | Lei L | 2015 | 2016 | Chongqing | College students | 554 | 6 |
| 70 | Mou SL | 2015 | 2016 | Chongqing | ICU medical staff | 265 | 4 |
| 71 | Yang CH | 2011-2013 | 2014 | Guangdong | ICU medical staff、Family members of the patients | 149/879 | 6 |
| 72 | Pan XM | 2010 | 2014 |  | Whole population | 174 | 4 |
| 73 | Zhang QX | 2016 | 2017 | Hunan | Transplant patients and their families | 426 | 6 |
| 74 | Zhang AH | 2014-2015 | 2015 |  | Whole population | 1, 074 | 7 |
| 75 | Zhang L |  | 2007 | Hunan | College students | 434 | 5 |
| 76 | Hu DM | 2013-2014 | 2015 | Liaoning, Guangdong | Medical staff | 373 | 5 |
| 77 | Luo AJ | 2012 | 2016 | Hunan | Whole population | 1, 085 | 7 |
| 78 | Yan JL | 2016 | 2018 | Beijing, Shanghai, Wuhan | Whole population | 2, 198 | 7 |
| 79 | Zhang JX | 2018 | 2018 | Nanjing | College students | 330 | 5 |
| 80 | Wang R | 2011 | 2013 | Hengyang | College students | 1, 943 | 5 |
| 81 | Wang LP | 2017 | 2018 | Guangdong | College students | 665 | 5 |
| 82 | Liang YQ |  | 2017 | Guangdong | College students | 925 | 5 |
| 83 | Gao Q | 2014 | 2015 | Zhejiang | College students | 626 | 6 |
| 84 | Zhan J |  | 2018 | Sichuan | Medical staff | 453 | 4 |
| 85 | Shan L | 2018 | 2018 | Sichuan | Whole population | 466 | 4 |
| 86 | Ji BX | 2017 | 2018 | Beijing | ICU medical staff | 106 | 6 |
| 87 | Huang F |  | 2013 | Hunan | Whole population | 988 | 5 |
| 88 | Du YY | 2017 | 2018 | Tianjin | College students | 431 | 6 |
| 89 | Liu BJ |  | 2017 | Beijing | Medical staff | 745 | 7 |
| 90 | Luo XY | 2014 | 2016 | Guangdong | Medical students | 1, 820 | 6 |
| 91 | Xiao DL | 2013 | 2015 | Hunan | College students | 1, 025 | 5 |
| 92 | Dong H |  | 2019 | Sichuan | ICU nurses | 321 | 5 |
| 93 | Xie JF | 2015 | 2017 | Nationwide | Nurses | 536 | 6 |
| 94 | Zhang H | 2017-2018 | 2018 | Nanjing | Medical staff、patients and family members | 443 | 6 |
| 95 | Geng ZX | 2017 | 2018 | Shandong | Whole population | 2, 511 | 5 |
| 96 | Zhou XN |  | 2016 | Beijing | Nurses | 303 | 5 |
| 97 | Jiao YL | 2012 | 2013 | Beijing | ICU nurses | 194 | 6 |
| 98 | Liu JQ |  | 2012 | Hunan | College students | 1, 943 | 4 |
| 99 | Zhou JL | 2010 | 2011 | Hunan | Baccalaureate nursing students | 341 | 5 |
| 100 | Wu HY | 2008 | 2008 | Sichuan, Wuxi | College students | 606 | 5 |
| 101 | Liang SM | 2018 | 2019 | Guangxi | Whole population | 499 | 5 |
| 102 | Hua J |  | 2019 | Xian | College students | 925 | 4 |
| 103 | Jia LN | 2011-2017 | 2019 | Xian | Whole population | 1, 480 | 4 |
| 104 | Li Y |  | 2019 | Anhui | Whole population | 1, 350 | 6 |
| 105 | Zeng H | 2018 | 2018 | Guangdong | Middle school students | 228 | 6 |
| 106 | He H | 2018 | 2018 | Sichuan | Medical staff | 200 | 5 |
| 107 | Dai AP | 2014 | 2015 | Hunan | Medical students | 425 | 6 |
| 108 | Zhang WY | 2015-2016 | 2017 | Tianjing | Whole population | 5, 868 | 6 |
| 109 | Wu Z | 2014-2016 | 2017 | Guangdong | Migrant workers | 660 | 6 |
| 110 | Xie QJ | 2016-2017 | 2018 | Guizhou | Whole population | 928 | 5 |
