# Supplementary material for: The general public new views on deceased organ donation in China
Source: Medicine (Baltimore). 2020 Dec 11;99(50):e23438. doi: 10.1097/MD.0000000000023438 (PMC7738062; doi:10.1097/MD.0000000000023438)

Supplemental Digital Content (SDC 4)

**The general public new views on** **deceased organ donation in China**

*Xiaoshan Li, PhD, Junyan Miao, BM, Rong Gao, PhD*

**SDC 4. The sources of the knowledge about organ donation of Chinese people.**


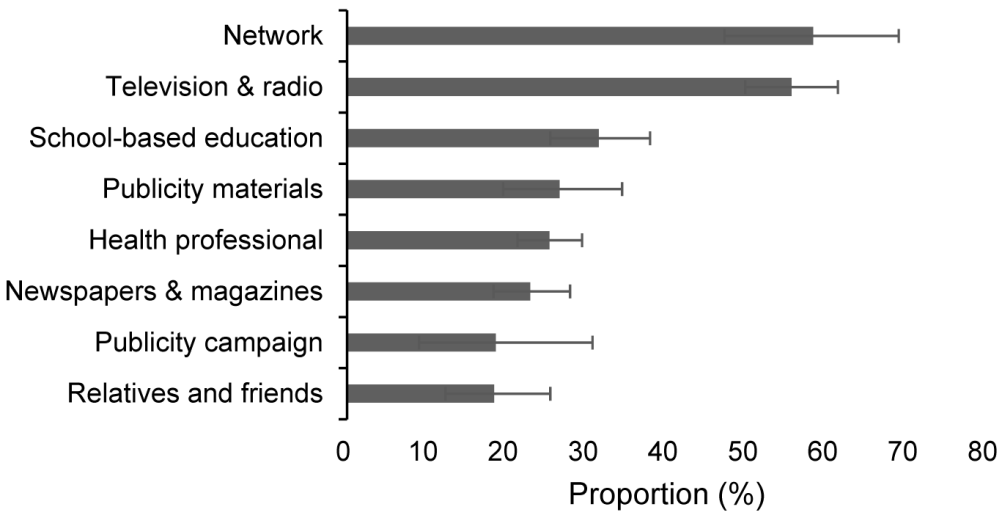

Supplement: Supplemental Digital Content [file medi-99-e23438-s004.docx]
