# Supplementary material for: The general public new views on deceased organ donation in China
Source: Medicine (Baltimore). 2020 Dec 11;99(50):e23438. doi: 10.1097/MD.0000000000023438 (PMC7738062; doi:10.1097/MD.0000000000023438)

Supplemental Digital Content (SDC 6)

**The general public new views on** **deceased organ donation in China**

*Xiaoshan Li, PhD, Junyan Miao, BM, Rong Gao, PhD*

**SDC 6. Willingness to posthumous organ donation with different genders**

There was no significant difference between the males and females for willingness to donate organs. Male (50.6%, 45.5- 55.8) and female (49.4%, 44.2-54.6) had the similar proportion of willing to donate their own organs, and the combined odds ratio was 1.10 (1.00-1.21), P= 0.05). Similarly, male (50.7%, 44.4-57.0) and female (49.3%, 43.0-55.6) had the similar proportion of refusing to donate organs, and the combined odds ratio was 0.93 (0.83-1.04), P=0.222.

**SDC 6. The willingness to posthumous organ donation with different genders.**


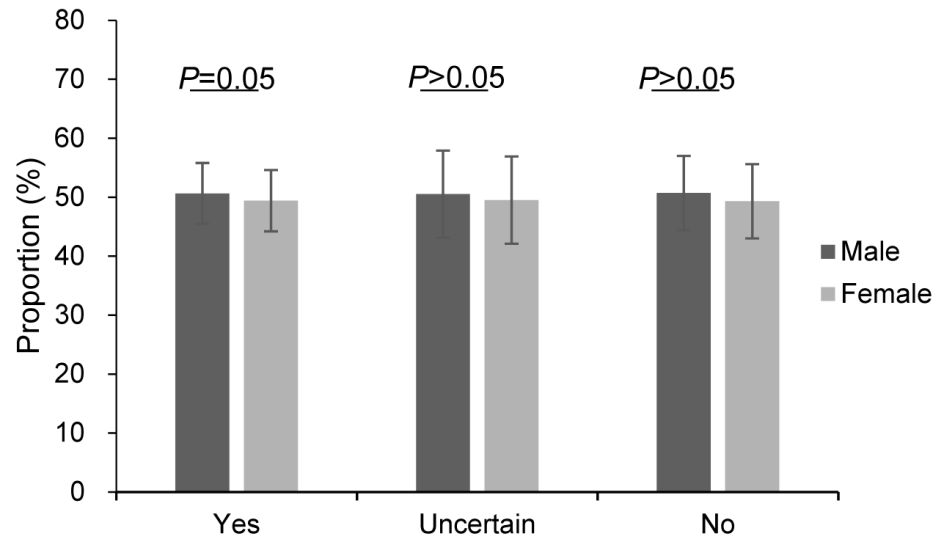

Supplement: Supplemental Digital Content [file medi-99-e23438-s006.docx]
